# RETRACTION: STUB1 Suppresseses Tumorigenesis and Chemoresistance Through Antagonizing YAP1 Signaling

**DOI:** 10.1111/cas.16307

**Published:** 2024-08-29

**RETRACTION**: D.‐E Tang, Y. Dai, L.‐W. Lin, Y. Xu, D.‐Z. Liu, X.‐P. Hong, H.‐W. Jiang, and S.‐H. Xu, “STUB1 Suppresseses Tumorigenesis and Chemoresistance Through Antagonizing YAP1 Signaling,” *Cancer Science* 110, no. 10 (2019): 3145–3156, https://doi.org/10.1111/cas.14166.

The above article, published online on 8 August 2019 in Wiley Online Library (wileyonlinelibrary.com), has been retracted by agreement between the journal Editor‐in‐Chief, Masanori Hatakeyama; the Japanese Cancer Association; and John Wiley & Sons Australia, Ltd.

The retraction has been agreed due to concerns raised by third parties on the data presented in the article. Specifically, the article shows results from two unknown/unverifiable cell lines, BSG‐823 and BGC‐803. The corresponding author acknowledged that both cell lines may correspond to the cell line BGC‐823 and stated that a typographical mistake was made by the provider in labeling the samples. However, these cell lines have been used as distinct and independent, raising severe concerns on the overall accuracy of the full body of data; the authors failed to comply to our request to provide raw data. Finally, several cell lines used in this study have been reported as problematic (SGC‐7901,^1^, ^2^ MGC‐803,^3^, ^4^ and MKN‐28^5^). For these reasons, the editors no longer have trust in the reliability of the data and consider the conclusions of this article to be invalid. The authors have been informed of the decision of retraction. Authors Yong Dai and Haowu Jiang state that they did not directly participate in the experiments conducted for the study and were unaware of its submission; they agree with the decision of retraction considering the detected issues. The corresponding author Song‐Hui Xu states that all authors have been duly informed of the submission of the article and agrees with the decision of retraction. The remaining co‐authors were unavailable for a final confirmation.
